# Feasibility of a computer-assisted social network motivational interviewing intervention for substance use and HIV risk behaviors for housing first residents

**DOI:** 10.1186/s13722-016-0061-x

**Published:** 2016-09-07

**Authors:** Karen Chan Osilla, David P. Kennedy, Sarah B. Hunter, Ervant Maksabedian

**Affiliations:** RAND Corporation, 1776 Main Street, PO Box 2138, Santa Monica, CA 90407-2138 USA

**Keywords:** Social network intervention, HIV risk behaviors, Data visualization, Alcohol and other drug use, Homelessness, Housing First, Motivational interviewing, EgoWeb

## Abstract

**Background:**

Social networks play positive and negative roles in the lives of homeless people influencing their alcohol and/or other drug (AOD) and HIV risk behaviors.

**Methods:**

We developed a four-session computer-assisted social network motivational interviewing intervention for homeless adults transitioning into housing. We examined the acceptability of the intervention among staff and residents at an organization that provides permanent supportive housing through iterative rounds of beta testing. Staff were 3 men and 3 women who were residential support staff (i.e., case managers and administrators). Residents were 8 men (7 African American, 1 Hispanic) and 3 women (2 African American, 1 Hispanic) who had histories of AOD and HIV risk behaviors. We conducted a focus group with staff who gave input on how to improve the delivery of the intervention to enhance understanding and receptivity among new residents. We conducted semi-structured qualitative interviews and collected self-report satisfaction data from residents.

**Results:**

Three themes emerged over the course of the resident interviews. Residents reported that the intervention was helpful in discussing their social network, that seeing the visualizations was more impactful than just talking about their network, and that the intervention prompted thoughts about changing their AOD use and HIV risk networks.

**Conclusions:**

This study is the first of its kind that has developed, with input from Housing First staff and residents, a motivational interviewing intervention that targets both the structure and composition of one’s social network. These results suggest that providing visual network feedback with a guided motivational interviewing discussion is a promising approach to supporting network change.

*ClinicalTrials.gov Identifier* NCT02140359

## Background

Substance use disorders and HIV infection are interrelated public health problems facing the homeless. An estimated 30–50 % of homeless adults experience alcohol and/or drug (AOD) use disorders [1, 2], and homeless persons have been found to have rates of HIV infection 3–9 times greater than those with stable housing [3]. While AOD use is a leading cause of homelessness, AOD use is exacerbated by the stress of being homeless and exposure to other people who use AODs [2, 4, 5].

Social networks play positive and negative roles in the lives of homeless people [6–8]. Social networks—naturally occurring groups of people—can influence an individual’s health and behaviors through social comparison, social sanctions and rewards, flows of information, support and resources, stress reduction, and socialization [9–12]. In the context of AOD and HIV risk behaviors, social networks can increase AOD use and HIV risk among those who are homeless, but also facilitate entry into AOD recovery programs and other healthy lifestyle changes [2, 4, 5, 13–16]. Continuous, recent homelessness is associated with the amount of AOD and HIV risk behaviors in social networks while total time spent homeless over a lifetime is associated with less dense and more disconnected networks (e.g., more isolated network members) [17]. Another study found that homeless individuals with co-occurring mental illness and substance use disorders experienced shrinking social networks, which reduced interactions with people who influenced them to use AOD, but also increased their social isolation and reduced their access to positive social resources, such as social support [14]. Thus, developing interventions that focus on social networks may assist individuals in supporting healthy behaviors.

Many social network interventions that target health improvement and behavior change utilize network analysis to identify techniques for spreading an intervention’s impact throughout a group [18, 19]. Common techniques include identifying key individuals or sets of individuals (e.g., those most central to the network, those most popular) to spread the intervention or modifying links among members of a group to make the intervention spread much more efficiently [19]. Other social network intervention approaches that target AOD behavior change primarily promote modifications to network composition (i.e., the quality and type of individuals in a network; removing substance users from the network) [20–28]. These interventions do not address the *structure* of social networks (i.e., relationship between network members; “Do people in your network interact with each other? How often have these two people interacted?”).

Addressing changes in network structure may be particularly important to homeless individuals transitioning into housing. Removing someone who drinks from a network is much easier if the person is disconnected from the rest of the network compared to someone who is highly interconnected. How these people are connected to each other (e.g., Are their new neighbors connected to their high-risk street contacts?) may impact how well they are able to negotiate this change. An intervention that focuses on both network composition (e.g., people they interact with that use AODs) and structure (e.g., people in one’s network who could meet one another to form a new support group) may help individuals make informed choices about their social interactions. To our knowledge, there are no interventions that take into account both compositional and structural characteristics of social networks targeting homeless individuals transitioning to housing.

The style in which network information is conveyed may be as important as the content itself. Motivational interviewing (MI) is a conversational style that is often used by facilitators conducting interventions that target AOD and risk behaviors. A facilitator that uses MI is collaborative and nonjudgmental, and focuses on strengthening the client’s own motivation and commitment to change [29]. The four processes of MI emphasize client engagement (establishing a helpful relationship, understanding barriers and reasons to change), focusing (identifying change area, and setting an agenda), evocation (eliciting the client’s motivation to change and building their self-efficacy), and planning (developing a commitment to change and formulating an action plan). We are aware of one recently developed MI intervention enhanced with a social network component that found that female adolescents who received the intervention had fewer AOD and HIV risk behaviors compared to those who did not receive the intervention at 1 month follow-up [27]. In this intervention, about 5 min were spent describing each of the network members the teens named and their association with substance use risk and support/encouragement. To our knowledge, visualizations were not presented, the intervention was developed for and tested with a limited sample of participants (i.e., female adolescents), and did not address network structure.

The current intervention extends this previous work by developing a computer-assisted social network intervention for homeless adults transitioning into housing. The intervention is computer or tablet-assisted so that a facilitator can collect personal network information from the participant, show visualizations of their social network immediately afterwards, and discuss potential areas of change using MI. The current paper describes the acceptability (likes/dislikes, ease of use, and helpfulness) of the intervention among residential support staff and formerly homeless people with histories of AOD and HIV risk behaviors.

## Methods

### Setting

This study was conducted in collaboration with Skid Row Housing Trust (SRHT), one of the largest Housing First providers in Los Angeles County. SRHT manages 22 buildings with over 1700 individual units, many of which provide housing plus support to residents (i.e., permanent supportive housing). Housing First programs provide housing without requiring AOD abstinence for new residents [30–32]. Studies have demonstrated that HF residents have similar [33] or improved [32, 34] AOD outcomes after 1–2 years compared to residents who receive AOD treatment first, and reduced health service expenses compared to those on waiting lists [35]. The current Housing First program provides permanent supportive housing (PSH), which are housing units that are supported by the U.S. Department of Housing and Urban Development (HUD). To be eligible for a PSH unit, an individual must meet the definition of chronic homelessness (i.e., an individual with a disabling condition who has been continuously homeless for a year or more, or has had at least 4 episodes of homelessness in the past 3 years).

### Participants and procedures

#### Overview

We conducted a 4-step iterative process to develop and evaluate the acceptability of the intervention. First, we conducted a focus group with staff (n = 6) to show them a draft of our intervention and discuss how residents might respond. Second, we role-played a first session with long-term residents (n = 6) who had resided in PSH for more than 1 year, and then conducted a focus group with these long-term residents to ascertain their acceptability of the session. Third, long-term residents and case managers nominated new residents (n = 5) with current AOD concerns, and we conducted a first session with each of them who provided us feedback on the acceptability of the intervention. Finally, a subset of these new residents (n = 3) then returned for a second session and provided feedback again. We revised the intervention iteratively between each step and obtained feedback on successive versions of the intervention.

#### Staff focus group

First, we recruited six residential support staff who were case managers, program managers, and administrators at SRHT. These staff members were nominated by the organization’s Resident Services Director for their diverse experience in assisting individuals entering permanent supportive housing. Participants were 3 men (2 Caucasian, 1 Hispanic) and 3 women (1 Caucasian, 1 African American, 1 Hispanic). Prior to beta-testing the intervention with residents, we developed a draft of the intervention to obtain staff feedback. The goals of the focus group were to discuss the logistics of the intervention process and obtain feedback about how they thought residents would respond to the intervention. We described the structure and content of the intervention including how the intervention visualizations would look, the intervention schedule, and how we would use tablets to deliver the intervention. We then role-played a mock intervention session, reviewed each of the intervention visualizations for feedback, and explored if the wording or visualizations were difficult to understand or may present problems if used in an intervention session with a resident. We requested their feedback on the language, structure, and presentation. Staff spoke from their professional capacity and verbally consented to the group discussion, which was audio taped and later transcribed.

#### Resident data collection

After the staff focus group session, we then conducted 3 rounds of beta testing (1 round with long-term residents and 2 rounds with new residents). First, we conducted individual interviews and then a focus group with long-term residents (n = 6) who had resided in SRHT for more than 1 year and residents with less PSH experience. These long-term residents were also peer advocates (i.e., employed by the housing provider to provide support to other residents) and had close contact with many new SRHT residents, and had past experience transitioning to PSH from homelessness. These residents completed a consent-to-contact form that allowed research staff to contact them by phone to schedule an in-person session. Long-term residents included 5 African American men and 1 Hispanic woman. All six long-term residents agreed to participate. At their session, each resident was asked to do three things. First, each resident participated in a role-play with one of the research staff members. Then, each resident was asked to role-play a new resident with risky AOD use and/or sexual risk behaviors while the research staff member facilitated one intervention session. After the session, the resident was asked to provide feedback about their experience. Finally, we conducted a focus group with all the long-term residents to gather collective feedback including the strengths and weaknesses of the intervention, and their perception of the acceptability and feasibility of the intervention for use with new residents. Participants were paid $25 for their participation.

We then revised the intervention by incorporating feedback from the long-term residents and conducted two rounds of individual interviews with new residents (n = 5) who had recently entered housing (< 6 months). These residents were nominated by long-term residents and case managers because of their previous or current AOD and/or HIV risk behaviors. All new residents that were nominated expressed interest by completing a consent-to-contact form. Participants included 3 men (2 African American, 1 Hispanic) and 2 women (2 African American). In the first round of beta-testing, we conducted the first intervention session with 5 participants, interviewed each participant after the session, and then asked them to complete a satisfaction survey. These sessions lasted between 45 and 60 min. After receiving this round of feedback, we revised the intervention, and then conducted a second intervention session with 3 of the 5 participants (we were unable to schedule the remaining 2 within our timeframe). Afterwards, we also conducted a debriefing interview with each participant and asked them to complete the satisfaction survey. A total of 3 facilitators led the Motivational Network Intervention (MNI) sessions with these residents, and the debriefing interviews were led by a different person by phone or in-person. The main purpose of this last round of beta-testing was to test the technology linking the sessions (e.g., if goals generated from the first session displayed correctly in the second session). All interviews were audio taped and followed a written protocol of open-ended questions. Participants were paid $25 per session, for a total of up to $50 for two sessions. All procedures were approved by the researchers’ Institutional Review Board.

### Intervention conceptual framework

The proposed Motivational Network Intervention (MNI) is grounded in social network theories (complex systems and social capital theories) and theories central to the MI approach [36]. The MNI targets social network structure and composition and how these are related to high-risk behaviors, such as AOD use and HIV risk behavior. Complex systems and social capital theories as applied to social networks assume that a set of social relationships between individuals in a group has emergent properties that would not be apparent in an examination of the individual parts of a larger social system [37–39] and that this system can produce positive or negative impacts on behavior [40]. These theories also suggest that changes made in one area of the system may have effects that flow throughout the rest of the system and that approaches to change should consider the potential impact on the whole system [39]. The theory of self-determination emphasizes individual autonomy and innate capacity for growth and change [41, 42]. Self-efficacy theory indicates that people with more confidence in their ability to change their behavior are more likely to change [43]. These theories are consistent with the style of MI that focuses on emphasizing an individual’s autonomy and building intrinsic motivation [29]. Together, these theories suggest that an intervention that presents residents with personalized network information using a MI style may empower them to change their social environment leading to changes in their behaviors.

### Intervention

Ultimately, we developed a computer-assisted intervention and a facilitator’s guidebook that assists facilitators in delivering the intervention content in concert with the computer or tablet-assisted material. Consistent with the MI approach, the intervention was designed to accommodate individuals at varying levels of readiness to change including those who were not ready to change their risk behaviors and those who were ready. The MNI consists of 4 total sessions where each session is spaced about 2 weeks apart. We decided to develop a 4-session intervention to allow residents enough time to engage in network change strategies and for residents to see changes in their social networks between sessions. After developing the intervention, we beta-tested only the first two sessions with participants because the content of all the sessions was nearly identical and we primarily wanted to evaluate the content and see how repeated sessions (e.g., discussing the visualizations in sessions 1 and 2) affected the participant. The technology successfully worked during our last round of beta-testing and we did not receive any new suggestions for changing the intervention content, so we decided to stop our beta-testing. We also reasoned that following a client across the 4 sessions was more appropriate for our pilot study where we intend on evaluating the efficacy of the intervention.

Each MNI session lasted approximately 30 min and consisted of two parts: (1) A network interview with closed-ended network questions that covered the time period since their last interview (e.g., about 2 weeks) and, (2) a discussion of network visualizations facilitated by using MI. Structured network interview questions were open-ended to generate names of people in their network (e.g., “List 10 people you have interacted with in the past 2 weeks”), network composition (e.g., “How likely will (Alter 1) use alcohol or drugs in the next 2 weeks?” “Did you ever drink more alcohol than you wanted with (Alter 1)?”), and network structure questions (e.g., “Does (Alter 1) know (Alter 2)?”). Answers to these questions provided raw data to generate network visualizations. One advantage to the electronic interface was that questions could be skipped for any alters who were mentioned in previous interviews to avoid re-asking for information that does not change between interviews. For all alters named, facilitators asked participants a series of questions rating their recent relationship with the alter. For example, questions included how often they interacted, AOD use with the alter, sexual relations with the alter, and their supportive or negative interactions with the alters.

**Fig. 1:**
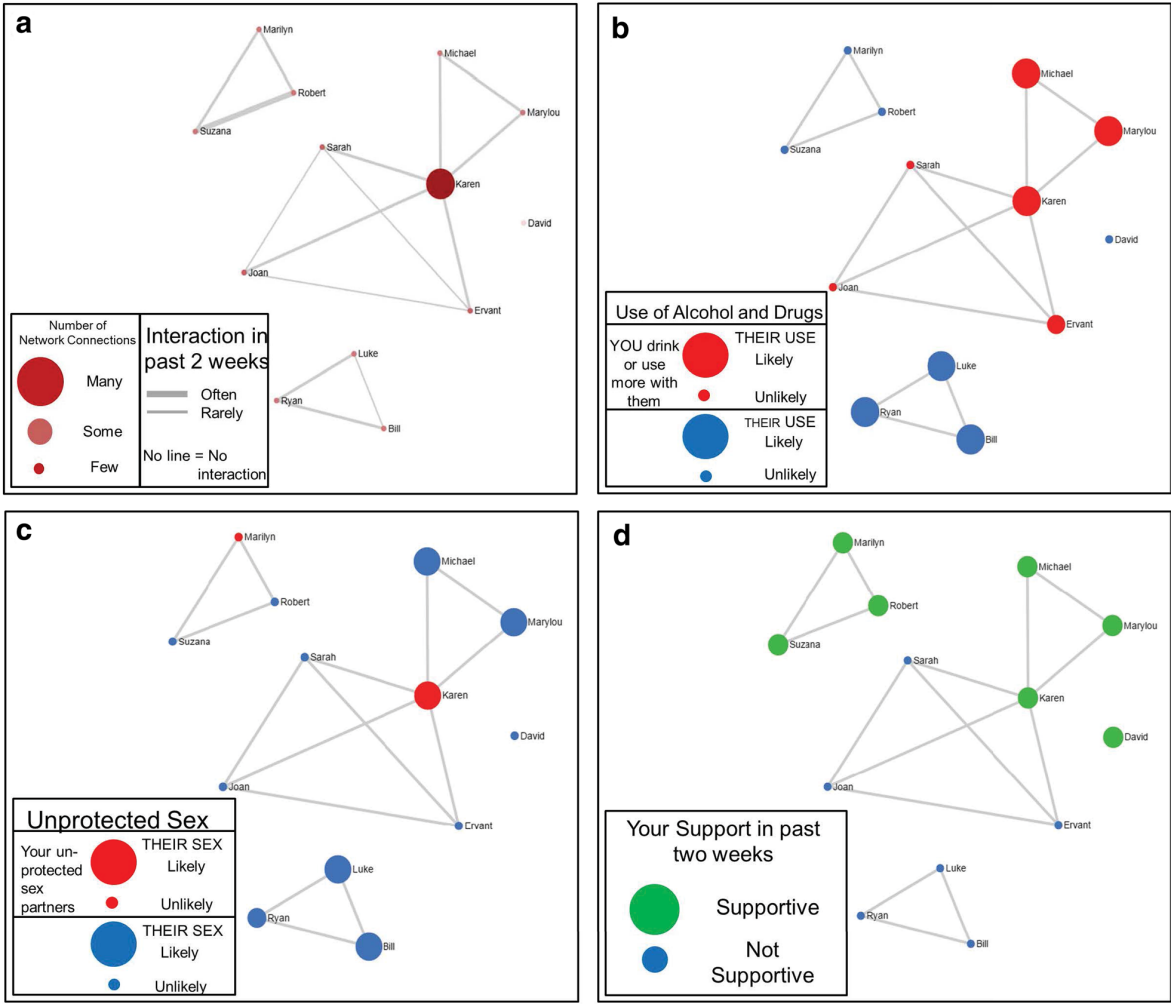
Example figures from hypothetical MNI session. Network contacts are represented by *circles* (graph “nodes”) and *lines* between nodes represent network contacts who interacted with each other in the past 2 weeks. The layout of the nodes, generated with the Fruchterman–Reingold force-directed placement algorithm highlights structural characteristics of the network, such as isolates (completely disconnected nodes) and components (a set of nodes tied together but disconnected from other nodes). The structural layout is consistent across the 4 diagrams. The figure in the *upper left* (**a**) uses node color and size, and line thickness to highlight other characteristics of the network structure, including the centrality of network actors (depicted by larger and darker nodes) and stronger relationship ties between actors (highlighted with *thicker lines*). The other figures use node size and color to highlight network composition. The figure in the *upper right* (**b**) highlights the likelihood of AOD use by network members with size (larger = likely, smaller = unlikely) and increased resident use when with network member by color (*red* drink or use more drugs with, and *blue* typical use). The figure in the *lower left* (**c**) highlights perceived risky sex by network members with node size (larger = likely, smaller = unlikely) and unprotected sex with network members with color (*red* had unprotected sex with, and *blue* did not have unprotected sex with). The figure in the *lower right hand* (**d**) depicts supportive network members with size and color (*large and green* supportive, *small and blue* not supportive)

Once all network questions were asked and answered, facilitators led a discussion with the participant about the participant’s social network in a MI style. They showed the participant a series of 4 network visualizations customized for the participant. The content of these visualizations were identical across the 4 sessions, but the “look” of the visualizations could change at each session depending on how much the participant’s network changed. Figure 1 depicts examples of the 4 visualizations for a hypothetical intervention participant. Network contacts are represented by circles (nodes) and lines between nodes represent network contacts who interacted with each other in the past 2 weeks. The network display uses a “spring embedding” visualization algorithm [44], which renders the array of connections among the nodes in two dimensional space, placing people who know each other and have similar ties to other network members close together, and people who do not further apart. The distribution of these nodes and lines highlights structural features of the network such as isolates (completely disconnected nodes) and components (a set of nodes tied together but disconnected from other nodes).

The diagram labeled (a) indicates the names of people that the participant reports interacting with in the past 2 weeks and highlights structural features of the social network by node size, color, and line thickness. The larger nodes signify the people that the participant reports know a lot of other people in the network (i.e. “degree centrality” [45, 46]), while the thicker lines between nodes denote the people that the participant reports have interacted frequently with each other. The 3 remaining visualizations in Fig. 1 represent the same network while highlighting different compositional characteristics using different node colors and sizes. The diagram labeled (b) highlights AOD use in the network with larger nodes highlighting people who are likely to use AOD in the next 2 weeks, and red nodes showing people the participant reports using AOD with in the past 2 weeks. The diagram labeled (c) uses node size to highlight the people who the participant reports are likely to have unprotected sex in the next 2 weeks (bigger nodes) and node color to denote who the participant reports having unprotected sex with (red nodes). The final visualization labeled (d) depicts network contacts whom the respondent rated as supportive with larger, green nodes (vs. smaller, blue nodes).

As each network diagram is displayed and discussed, the intervention facilitators explored the pros and cons of participants’ current social network composition and structure, and discussed their readiness, willingness and confidence to change risky aspects about their networks (e.g., “Tell me which of these people affect your drinking the most. What do you think about them?”). Facilitators also looked for opportunities to encourage discussion of strategies for positive behavior change. For example, if the participant has few supportive people in their network, the intervention facilitator can ask the participant, “Is there someone you haven’t named who you would like to interact with more? What are some steps you can take to interact with that person in the next 2 weeks?” In addition, facilitators asked participants to rate how willing they were to change their AOD use and sexual behaviors (on a scale from 1 to 10 where “1” is not willing and “10” is very willing) when they discussed these respective network visualizations. The electronic tool included a large text box on each visualization screen and a node-level note annotation interface for recording statements from participants about making positive behavioral changes during the discussions. After discussion of the 4 visualizations, participants were asked to list some goals related to their AOD use or sexual behaviors that they would try to achieve over the next 2 weeks before their next session. As stated earlier, the four sessions were nearly identical with two exceptions. First, session 1 did not include a review of the previous session’s graph. Second, session 4 included additional questions regarding future goals with their AOD and sexual risk behaviors, and how to prevent relapse if they have changed.

### Measures

#### Interview protocol

To assess acceptability of the intervention among staff and residents, we asked about two main categories: Likes/dislikes and helpfulness in changing AOD and HIV risk behaviors to the target population of new PSH residents. To assess likes and dislikes, we asked participants about their general thoughts about the session (e.g., “What did you like/dislike? What did you think of the visualizations?”), how easy or difficult they perceived the questions to answer (e.g., “What was it like talking about the 10 or 15 people in your life right now and how it’s been going?”). To assess helpfulness, we asked how the intervention might impact residents with AOD and HIV risk behaviors (e.g., “How do you think new residents will react to getting this information? How does this information affect your social network?”). Staff were also asked about the logistics of delivering the intervention (timing, frequency, and mode).

#### Satisfaction survey

After each interview, new resident participants (n = 5) completed a 20-item self-report satisfaction survey that asked about four areas. These included 6 questions about their overall impressions (e.g., “The different activities we did in the session were helpful.”), 4 questions about the social network visualizations (e.g., “How helpful was the network picture highlighting alcohol and drug use?”), 4 questions about how the intervention might affect new residents (e.g., “I feel that the things I did in the session will help new residents to make the changes that they want.”), and 6 questions about the facilitator (e.g., “The facilitator valued my opinion.”). Participants were asked to rate each item on a 5-point Likert scale, with a higher score representing higher acceptability and satisfaction. Similar satisfaction questions have been used in prior research [47–49]. The 3 resident participants who completed a second intervention session were also asked to complete another round of the satisfaction survey.

### Analyses

#### Qualitative analyses

The qualitative procedures and analyses for the staff focus group and resident interview were adapted from previous studies with this population and type of intervention [5, 48–56]. First, all audiotaped interviews were transcribed. Second, the written transcripts from the interviews were imported into the qualitative analysis software Dedoose [57] for analytic purposes. Third, 3 researchers (KCO, DPK, and EM) independently reviewed the transcripts in Dedoose to identify, characterize, and categorize the key themes. The purpose of this review was to identify, label, and group together key points that spoke to what participants liked or disliked about the intervention and whether they found the intervention helpful in thinking about reducing AOD and HIV risk behaviors. Following grounded theory analyses [58], key points with similar concepts were grouped together into a category if said several times by different participants over time (e.g., comments that the visualizations were insightful). The 3 coders then tagged quotes illustrating each theme. Classic content analysis was used to identify quotes that fit each of the themes (e.g., visualizations were impactful) [59, 60]. After initial coding, team members reviewed the entire list of tagged quote excerpts, identified and discussed disagreements with initial coding, and then came to a consensus on a final set of themes. A final summary description of each theme was written into a codebook.

#### Quantitative analyses

We conducted descriptive analyses of the satisfaction data examining how participants rated the quality and their satisfaction with the session, social network visualizations, and facilitator. We also conducted descriptive analyses examining how participants thought the MNI would impact new residents.

## Results

### Staff focus group

Our staff focus group yielded three main findings regarding the language, structure, and presentation of the intervention. First, staff recommended we change certain wording (e.g., say “unprotected sex” instead of “risky sex”; query about oral sex in additional to vaginal and anal sex) to enhance resident understanding and engagement. Second, staff had mixed reactions about when we should start the intervention. Some recommended starting the first session 4 weeks or more after residents enter housing instead of within 2 weeks because they were worried that residents would not be honest about their risk behaviors, while other staff thought it may be easier to recruit clients to the study early on when they were motivated to enter housing. They recommended we clearly outline our rules about confidentiality to encourage honest reporting. Finally, staff had several positive comments about the visualizations stating they liked the colors of the nodes and the sizes of the circles to distinguish different people in their network. They stated that the visualizations may lower any defensiveness naturally engendered when discussing their substance use and sexual risk behaviors. They also recommended that we use computers versus tablets to deliver the intervention because the visualizations may be easier to see on a larger monitor. They recommended bigger fonts and surface area to see the intervention visualizations and the need to have a backup if internet connectivity was not available. Finally, staff recommended that we beta-test our intervention with long-term residents who were also peer advocates in addition to new residents because of their relevant experiences.

### Residents

We group the themes from both the long-term residents and new residents together because their feedback was similar. The three themes that emerged were that the intervention was helpful in discussing their social network, that seeing the visualizations was more impactful than just talking about their network, and that the intervention prompted thoughts about changing their AOD use and HIV risk networks. Each theme is described below. Table 1 elaborates on each theme by providing additional participant quotes.

**Table 1:** Themes from resident interviews

Theme	Participant quotes
Intervention was helpful in examining their social network	I thought it was awesome
	It was helpful to me also to stay motivated and stay positive
	It showed me the connection that one must have in order to stay focused. You can be connected to an awesome network, people that’s moving forward…and also you can be connected to a network that’s dying. So it is a network whether it’s good or bad…it’s just which one you choose to be connected to
	It helps you see who really around you is helping you, who is your support system, and how do you feel about your support system, and whether or not you’re going to change your daily behavior and/or interactions
Seeing the visualizations is more impactful than just talking about their network	Well, actually I see my support system. Visually I can see it. It’s different between thinking it and all that, but seeing it lets me know that this is correct
	It makes you see the pattern of your own life, and you visualize it, you know what I mean, it’s not just in your mind
	With a case manager you set goals, but this is better. It shows... your activities. You know, I can see who’s bad for me and who’s not bad for me
	Seeing it is different than just somebody telling you or talking about it. Seeing it makes it easier to understand
Intervention prompted thoughts about changing their AOD use and HIV risk networks	I need to not be up in their face, I need them not to be up in mine, because if I could stop smoking cocaine, I know I could slow down on my drinking. But it’s the environment that I be around, the environment that I be around, the people that be in my circle, and I be in their circle, I need to change that
	So as far as not drinking, I haven’t been going to see my friends who drink. And I’ve been meeting new friends, and hoping, you know, like non-drinking, and if I go for information, I call individuals that are in AA. I’m getting closer to that also
	If I surround myself with people that have my old mentality, it’s just going to keep me trapped in my same situation bringing me no type of change. So if I expand my surroundings, expand the people that I deal with, and cut out people that I know that I shouldn’t be dealing with, or that aren’t really beneficial to me

The first theme that participants frequently mentioned was how helpful the intervention was in discussing and examining the people currently in their lives. For example, one respondent said, “It made me think about who is in my life…who I interact with” and “It kind of shows you who you need to be with and who you don’t need to be with.” Some commented on how this insight helped them understand their own behaviors. For example, “I also see what I gravitate to more…which is good, because I can see what I’m doing.”

There were some negative comments in the early stages of the beta testing regarding the number of alters and type of alters that participants were being asked to name. As a result, we changed the instructions to add flexibility to the number of alters that participants are asked to name, as the participants indicated it may be challenging for some residents to generate 20 people that they had interacted with in the past 2 weeks and others suggested that 10 names might be too few to identify important relationships. Also, participants expressed concern that the instructions were too ambiguous and that they may name children that would not be relevant to their AOD and HIV risk behavior. For example, one participant said, “because if I had named ten other different people, you would have got a totally different read, a totally different understanding. So I guess that’s why I was kind of confused because I didn’t know what direction you was going, what basically you were trying to find out, what were you trying to find out about?” Therefore, we modified the instructions so that participants were prompted to mention at least 10 adults (up to 15) that they had interacted with in the past 2 weeks.

Second, when asked about their feedback about the visualizations, the majority of participants felt that *seeing* their networks was much different and more beneficial than merely talking about the people in their life. For example, one participant stated, “it’s easy to talk about, but when I see who I should be with, who I shouldn’t be with, it’s a different issue, so it makes more sense”, and another participant stated that they realized after seeing the visualization that “this [social network] circle is not going to work for me. You know, hearing about it is one thing, but seeing it is another.” Some participants also commented that the visualizations were easy to understand. One participant stated, “it’s a concrete way to see the big green circles are good, the big red ones are bad” and another participant stated, “The big circle I know for a fact there’s unprotected sex there”. Overall, the participants appeared to understand the purpose of using the visualizations to talk about social network change.

Finally, some residents who completed two sessions discussed how the session information helped them explore changes to their networks and/or their AOD or sexual risk behaviors. For example, one participant stated: “it showed me which ones I should be with, in case I need to, you know, if I’m trying to stop smoking, stop drinking, stop drugs, it kind of shows you who you need to be with and who you don’t need to be with". Another participant stated, “I see sobriety in the smallest circles, I see social [drinkers] in the medium circles…and then I see loss of control in the larger circles, and that’s why the [larger circles are] falling away from my network.” While we cannot conclude whether these changes were a result of the intervention itself, participants who reported change to their AOD and HIV risk behaviors consistently noted changes to their social networks. One participant talked about his network composition and how he “cut a lot of people out” and added “replacements” for the AOD-using individuals so that he could build a stronger support system. Another participant used the visualizations of her network structure to build her self-efficacy when stating, “…and by looking at having unprotected sex, how if a couple more lines would have been more connected, I would have been a little more scared, because I don’t know who’s sleeping with who. So two more lines and I’d have to run to the clinic.”

### Satisfaction survey

Participants rated the sessions very highly as ratings were between a 4 (agree) and 5 (strongly agree; 1-5 Likert scale) in all 4 domains. Participants were highly satisfied with the overall session. On average, they agreed or strongly agreed that they had left the session with a specific goal in mind about changing their AOD use habits, as well as their social networks, and had found the session activities helpful. Participants also highly rated the social network visualizations, reporting they agreed or strongly agreed that the pictures showing their interaction, social support, alcohol and drug use, and sex and condom use were helpful. They also agreed or strongly agreed that the facilitators were well trained, valued and respected the respondent’s opinions, and were helpful throughout the session. Finally, participants also thought the session would positively impact new residents.

## Discussion

Delivering a motivational social network intervention for individuals transitioning into Housing First programs was found to be both acceptable to staff and residents of these programs. An intervention that focuses on an individual’s social network appears especially important during this period, and for this population given findings that suggest that homeless individuals may have relatively small social networks with limited social support [14], and recent data that suggests risk behaviors such as AOD use and unprotected sex, may increase as one transitions from homelessness to supportive housing [16].

Findings from this study suggest that providing network visualizations based on a social network interview coupled with a guided discussion using a MI approach is perceived as both helpful and understandable. More specifically, staff gave input on how to improve the delivery of the intervention to enhance understanding and receptivity among new residents. Staff thought that the intervention would provide support to residents in making positive behavioral changes while transitioning from homelessness to supportive housing. In addition, resident participants overwhelmingly agreed that they thought being able to view their social network helped them better understand their personal relationships and its impact on their own behavior. Moreover, resident participants who engaged in more than one beta testing session reported that they had made behavioral changes as a result of the previous social network discussion. These results suggest that providing network visualizations with a guided MI discussion is a promising approach to supporting behavioral change.

Although the intervention tested in this study was specifically designed for homeless individuals transitioning into Housing First programs, we believe that a social network intervention that uses a MI approach could be helpful to other populations that need support for making a behavioral change. Presenting information about the structure and composition of one’s network prompts individuals to consider how people in their lives and the relationships among those people are relevant to their future behavior. MI, a therapeutic style that is especially helpful for resolving ambivalence about one’s behavior, has demonstrated effectiveness in reducing problematic behaviors, such as heavy drinking [61, 62]. Pairing a social network intervention with MI is a novel way in which to address how personal relationships may enhance or detract one from making healthy decisions. The intervention materials and techniques developed in this study are not specific to the homeless or Housing First programs and may be appropriate for testing in other settings.

The results presented herein represent the first phase of a clinical trial planning grant. More specifically, the findings are from iterative beta testing of the computer-assisted intervention with participants that are similar to the target population (i.e., individuals that are formerly homeless individuals as they transition to permanent supportive housing). Conducting beta testing as part of the initial intervention development is consistent with expert guidance on behavioral therapy research [63]. Next steps in the research of this intervention follow these guidelines, that is, a small pilot study where recruitment of a sample of homeless individuals that are transitioning to permanent supportive housing will be randomly assigned to receive the intervention or usual case management to explore the potential efficacy of the intervention. This pilot study [64] will be used to plan for a larger clinical trial if preliminary evidence of the intervention’s efficacy is established.

### Limitations

Our study has several limitations. The results presented herein are from a small purposive sample of Housing First staff and residents. The sample size is appropriate for beta testing where the goal is to collect in-depth feedback from potential users about the format of the intervention and their understanding of it. However, the study design does not allow us to make any inferential claims regarding the effectiveness of the intervention. Our findings were highly consistent across participants and therefore we saw little value in increasing the sample size. Also of note, some of the data were from a small sample of formerly homeless individuals living in project-based housing in a metropolitan area. It is possible that staff from different supportive housing programs and formerly homeless individuals living in other parts of the country (e.g., rural settings) or housing conditions (i.e., scattered sites) would have perceived the intervention differently. We acknowledge that the resident participants were living in project-based housing near large homeless encampments (i.e., Skid Row) which may make the transition to housing especially challenging and hence the need for an intervention that focuses on one’s social network more relevant. The beta testing met our goals of refining the intervention in preparation for a pilot study.

## Conclusions

In sum, this study is the first of its kind that has developed, with input from Housing First staff and residents, a motivational interviewing intervention that targets both the structure and composition of one’s social network. The purpose of the intervention is to reduce AOD and HIV risk behaviors among those transitioning to permanent supportive housing. Previous research suggests that this transition may serve as a critical time to intervene to prevent future risk. Our results show that the intervention was perceived as acceptable by staff and residents. More research is needed with a larger sample and longer time frame to explore the potential effectiveness of the intervention in reducing AOD and HIV risk behaviors.
